# Computed tomography perfusion ischemic core underestimation: a perfusion scotoma case

**DOI:** 10.1007/s10072-023-07139-3

**Published:** 2023-10-23

**Authors:** Giorgio Busto, Elisa Scola, Enrico Fainardi

**Affiliations:** 1 Department of Neuroradiology, Careggi University Hospital, Largo Piero Palagi 1, Florence, Italy; 2 Department of Experimental and Clinical Biomedical Sciences “Mario Serio”, Neuroradiology Unit, University of Florence, Florence, Italy

**Keywords:** CT perfusion, Stroke, Collaterals

**Fig. 1:**
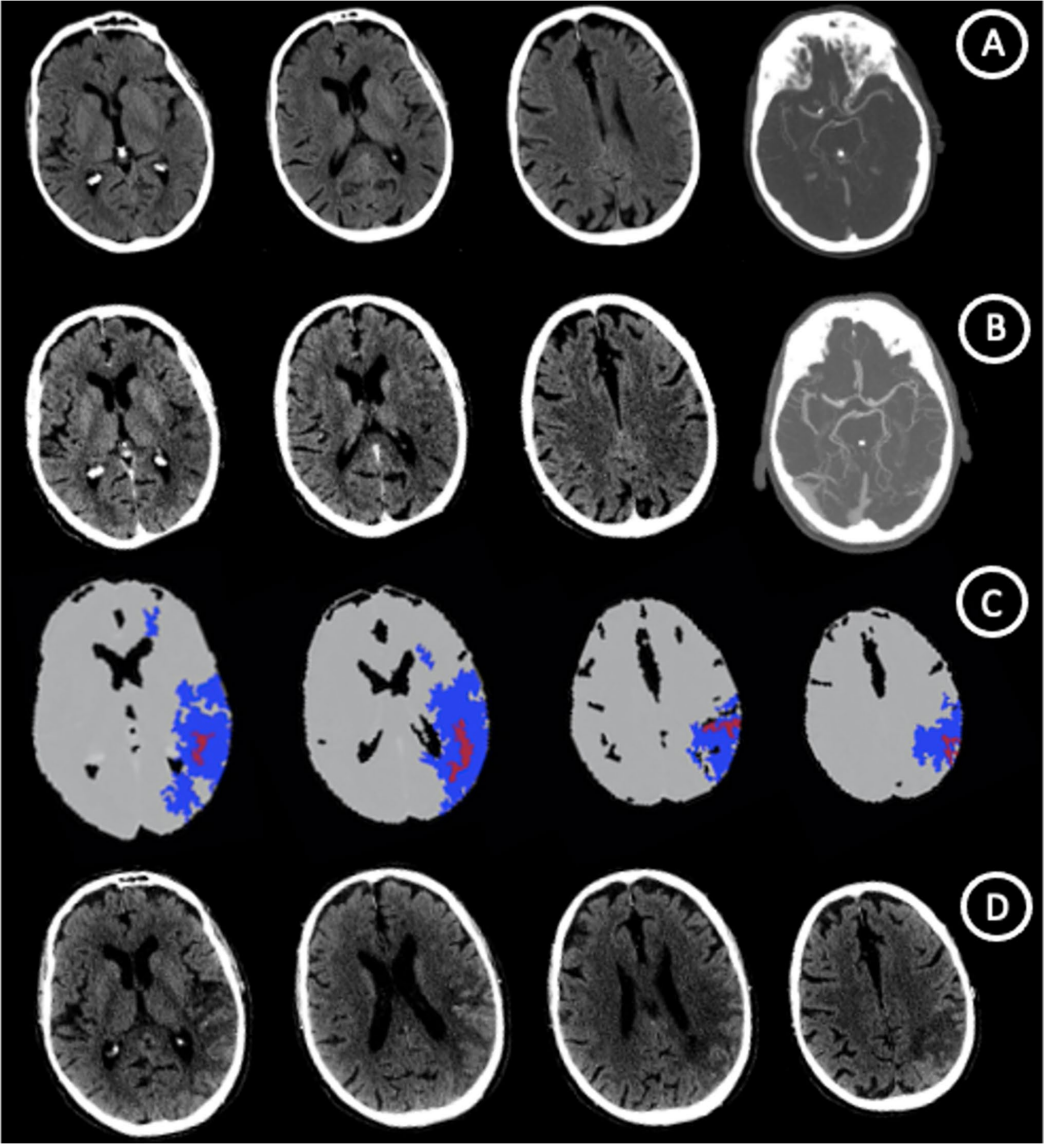
Neuroimaging study protocol in spoke and hub center: NCCT with ASPECT score of 6 and distal M1 cerebral artery occlusion. (**A**) NCCT, mCTA and CTP > 6 h from symptom onset with spontaneous recanalization and ischemic core underestimation due to luxury perfusion (**B**,**C**). NCCT at 3 days uphold a large ischemic core size (**D**)

An 82 yo man with aphasia and right-side weakness performed NCCT and single-phase CTA from 2.5 h from symptoms onset in spoke center that showed a large parenchymal hypodensity referring to acute ischemic stroke with ASPECT score of 6 (Insula, M2, M3, M6 segments) due to distal M1 cerebral artery occlusion. 3.5 h later, neuroimaging study protocol with NCCT, multi-phase CT-Angiography and CT-Perfusion in hub center was repeated. CT-Perfusion was realized with OLEA Perfusion Software that define total hypoperfused tissue with Tmax > 6 s and infarct core with rCBF < 40%. A spontaneous partial vascular recanalization with leptomeningeal vasodilatation occurred and CTP showed a little ischemic core (3,8 ml) and large penumbra (108,5 ml). 3 days after, NCCT uphold the ischemic core size. This case shows a typical Perfusion Scotoma with underestimation of ischemic core volume > 6 h from symptom onset due to early reperfusion from partial recanalization as well as luxury perfusion [1, 2] (Fig. 1).
